# Intracellular Zinc Modulates Cardiac Ryanodine Receptor-mediated Calcium Release*

**DOI:** 10.1074/jbc.M115.661280

**Published:** 2015-06-03

**Authors:** Jason Woodier, Richard D. Rainbow, Alan J. Stewart, Samantha J. Pitt

**Affiliations:** From the ‡ School of Medicine, University of St. Andrews, St. Andrews KY16 9TF, United Kingdom and; the § Department of Cardiovascular Sciences, University of Leicester, Clinical Sciences Wing, Glenfield General Hospital, Leicester LE3 9QP, United Kingdom

**Keywords:** calcium, excitation-contraction coupling (E-C coupling), heart failure, ryanodine receptor, zinc

## Abstract

Aberrant Zn^2+^ homeostasis is a hallmark of certain cardiomyopathies associated with altered contractile force. In this study, we addressed whether Zn^2+^ modulates cardiac ryanodine receptor gating and Ca^2+^ dynamics in isolated cardiomyocytes. We reveal that Zn^2+^ is a high affinity regulator of RyR2 displaying three modes of operation. Picomolar free Zn^2+^ concentrations potentiate RyR2 responses, but channel activation is still dependent on the presence of cytosolic Ca^2+^. At concentrations of free Zn^2+^ >1 nm, Zn^2+^ is the main activating ligand, and the dependence on Ca^2+^ is removed. Zn^2+^ is therefore a higher affinity activator of RyR2 than Ca^2+^. Millimolar levels of free Zn^2+^ were found to inhibit channel openings. In cardiomyocytes, consistent with our single channel results, we show that Zn^2+^ modulates both the frequency and amplitude of Ca^2+^ waves in a concentration-dependent manner and that physiological levels of Zn^2+^ elicit Ca^2+^ release in the absence of activating levels of cytosolic Ca^2+^. This highlights a new role for intracellular Zn^2+^ in shaping Ca^2+^ dynamics in cardiomyocytes through modulation of RyR2 gating.

## Teaser

**Background:** In heart failure, the release of calcium becomes erratic leading to the generation of arrhythmias. Dysregulated Zn^2+^ homeostasis occurs in chronic heart failure.

**Results:** Zn^2+^ can directly activate RyR2, removing the dependence of Ca^2+^ for channel activation.

**Conclusion:** Zn^2+^ shapes Ca^2+^ dynamics by directly interacting with and modulating RyR2 function.

**Significance:** This highlights a new role for Zn^2+^ in cardiac excitation-contraction coupling.

## Introduction

In cardiac muscle, the intracellular signal that triggers muscle contraction is thought to be a transient rise in intracellular Ca^2+^ that leads to the opening of Ca^2+^ release channels called type 2 ryanodine receptors (RyR2)^2^ on the sarcoplasmic reticulum (SR). The resulting release of Ca^2+^ into the cytosol causes movement of contractile myofibrils leading to cell contraction. Damaging changes in Ca^2+^ homeostasis are associated with heart failure, conduction abnormalities, and contractile dysfunction. Abnormal RyR2 function is recognized as an important component in the etiology of such disease states (1–3).

Recently, there has been much interest in the role of Zn^2+^ as an intracellular signaling molecule (4–6). Like Ca^2+^, intracellular Zn^2+^ is heavily buffered (7), and cellular Zn^2+^ homeostasis requires mechanisms that tightly control the uptake, storage, and distribution of Zn^2+^. This is achieved through the coordinated actions of metallothionein proteins and Zn^2+^ transporters (8–10).

In cardiomyocytes, the resting intracellular Zn^2+^ concentration is reported to be at picomolar levels (∼100 pm) (11, 12). During cardiac excitation-contraction coupling, intracellular Zn^2+^ concentrations are altered, and spatiotemporal fluctuations in free Zn^2+^ levels, including both Zn^2+^ transients and Zn^2+^ sparks, are suggested to be remarkably similar to those previously shown for Ca^2+^ (13). Other groups suggest that Zn^2+^ can permeate through the L-type channel with a greater affinity than Ca^2+^ but with a much lower permeability leading to a reduction in the inward current (14, 15). It has also been suggested that the SR can function as an intracellular store for Zn^2+^ alongside Ca^2+^ (6, 13).

Importantly aberrant Zn^2+^ homeostasis has been shown to be associated with cardiomyopathy including chronic heart failure, and myocardial damage as a result of dysregulated intracellular Ca^2+^ release, reduced cardiac contractility, and significantly prolonged rises of systolic Ca^2+^ (16–19). The potential role of Zn^2+^ in shaping intracellular Ca^2+^ release and regulating intracellular Ca^2+^ dynamics in heart however, is poorly characterized. Here we show that Zn^2+^ is a potent regulator of SR Ca^2+^ release through modulation of RyR2 channel function and that Zn^2+^ plays a key role in shaping intracellular Ca^2+^ dynamics important in cardiac excitation-contraction coupling.

## Experimental Procedures

### 

#### 

##### Reagents

Chemicals were AnalaR or the best equivalent grade from BDH Chemicals (Poole, UK) or Sigma-Aldrich. All solutions were made in deionized water, and those for use in bilayer experiments were filtered through a Millipore membrane filter (0.45-μm pore). 2,2′-(Ethylenedioxy)dianiline-*N*,*N*,*N′*,*N′*-tetraacetic acid (BAPTA) (Dorset, UK) and *N*,*N*,*N′*,*N′*-tetrakis(2-pyridylmethyl)ethylenediamine (TPEN) were from Sigma-Aldrich.

##### SR Vesicle Preparation and Planar Phospholipid Bilayer Techniques

Sheep hearts were obtained from a local abattoir, and heavy SR membrane vesicles were prepared and fused with planar phosphatidylethanolamine lipid bilayers, as described previously (20). Briefly, heavy SR vesicles were prepared as follows. Homogenized ventricular cardiac tissue from sheep was subjected to centrifugation at 6,500 × *g* followed by ultracentrifugation of the supernatant at 100,000 × *g*. The heavy SR membrane fraction was obtained from loading mixed membranes onto a discontinuous sucrose density gradient. Heavy SR was collected, snap frozen in liquid N_2_, and stored at −80 °C. As reported previously, SR vesicles fused in a fixed orientation such that the *cis*-chamber corresponded to the cytosolic face of the channel and the *trans*-chamber to the SR lumen (21, 22). The *trans*-chamber was held at ground and the *cis*-chamber at potentials relative to ground. After fusion, the *cis*-chamber was perfused with 250 mm HEPES, 80 mm Tris, and 10 μm free Ca^2+^ (pH 7.2). The *trans*-chamber was perfused with 250 mm glutamic acid and 10 mm HEPES (pH to 7.2) with Ca(OH)_2_ (concentration of free Ca^2+^ ∼50 mm). The identity of RyR2 was confirmed by the single-channel conductance, and the number of channels gating in the bilayer was assessed by the application of caffeine at the end of the experiment. Zn^2+^ was added as ZnCl_2_ to the *cis*-chamber at the required concentration from an appropriate stock solution. 100 mm stock solution of ZnCl_2_ was prepared in 0.029 m HCl, and serial ZnCl_2_ dilutions were made in a Tris/HEPES buffer which contained, 250 mm HEPES, 80 mm Tris, and 10 μm free Ca^2+^ (pH 7.2) to make appropriate stock solutions. The concentration of each Zn^2+^ stock was further assessed using a pZn meter (Neurobiotex.com, Galveston, TX) as previously described (23) to confirm the accuracy of our serial dilutions. The lowest concentration Zn^2+^ stock made was 10 nm. The addition of 100 μm ZnCl_2_ to our Tris/HEPES buffer had no significant effect on the pH (pH was 7.22 ± 0.014 before and 7.21 ± 0.016 after the addition of 100 μm ZnCl_2_; *n* = 3)_._ Special care was taken when preparing Zn^2+^ stock solutions. All glassware was prewashed with 100 nm TPEN and then rinsed carefully with MilliQ water passed through a Chelex Resin (Bio-Rad). The use of colored pipette tips was also avoided.

Experiments were carried out at room temperature (22 ± 2 °C). The concentration of free Ca^2+^ and pH of all of our solutions were determined using a Ca^2+^ electrode and a pH electrode (Hanna Instruments, Bedfordshire, UK) as previously described (20). Our measurements showed that the addition of Zn^2+^ ≤ 100 μm had no significant effect on the concentration of free Ca^2+^ ([Ca^2+^] was 10 ± 3.8 μm in the absence of Zn^2+^ and 10 ± 3.2 μm in the presence of 100 μm Zn^2+^ (*n* = 3). The addition of 1 mm BAPTA reduced the free Ca^2+^ concentration from 10 ± 2 μm to 10 ± 3.8 nm (*n* = 3). Subsequent addition of ZnCl_2_ (≤100 μm) had no significant effect on the measured free Ca^2+^ concentration (10 ± 3.2 nm; *n* = 3) under these conditions. The total concentration of Zn^2+^ in all of our recording solutions was assessed using inductively coupled plasma optical emission spectrometry (ICP-OES) (University of Edinburgh, Grant Institute, commercial facility) and shown to be below the detectable limit (1 ppb). Free Zn^2+^ was also assessed using a pZn meter and calculated to be ∼9 pm. The addition of concentrations of Ca^2+^ ≤ 100 μm had no significant effect on the concentration of free Zn^2+^ ([Zn^2+^] was 9 ± 7 pm in the presence of 10 μm Ca^2+^ and 10 ± 6 pm when Ca^2+^ was raised to 100 μm; *n* = *3*). The calculated free Zn^2+^ concentration following addition of 1 mm cytosolic BAPTA was first estimated using the MaxChelator program and then assessed by actual measurements of free Zn^2+^ using a pZn meter. When the starting free Ca^2+^ concentration was 10 μm, the addition of 1 mm BAPTA had no significant effect on free Zn^2+^ levels.

##### Single Channel Recording and Analysis

Single-channel currents were monitored under voltage-clamp conditions using a BC-525C amplifier (Warner Instruments, Harvard Instruments). Channel recordings were low pass filtered at 10 kHz with a 4-pole Bessel filter, digitized at 100 kHz using a National Instruments acquisition interface (NIDAQ-MX; National Instruments, Austin, TX), and recorded on a computer hard drive using WinEDR 3.05 software (John Dempster, University of Strathclyde, Glasgow, UK). The recordings were subsequently filtered at 800 Hz (−3 dB) using a low pass digital filter implemented in WinEDR 3.05. Channel events were detected by the 50% threshold method (24) using TAC 4.2.0 software (Bruxton Corporation, Seattle, WA). Open probability (*P*_o_) and lifetime distributions were calculated from 3 min of continuous recording using TACfit 4.2.0 software (Bruxton Corporation). Lifetime analysis was carried out only when a single channel was incorporated into the bilayer. Individual lifetimes were fitted to a probability density function using the method of maximum likelihood (24). Lifetimes of <1 ms were not fully resolved under the conditions of data acquisition described here and were therefore excluded from the fitting procedure. A missed events correction was applied as previously described (25).

##### Ca^2+^ Waves

Adult male Wistar rats (300–400 g) were killed by concussion followed by cervical dislocation. The care and sacrifice of the animals conformed to the requirements of Directive 2010/63/EU of the European Parliament.

##### Isolation of Cardiomyocytes

All extracellular solutions were based on a modified Tyrode's solution. The basic solution contained, 135 mm NaCl, 5 mm KCl, 330 μm NaH_2_PO_4_, 5 mm glucose, 5 mm sodium pyruvate, 10 mm HEPES, 1 mm MgCl_2_, and 2 mm CaCl_2_ (pH 7.4). Nominally Ca^2+^-free Tyrode's solution used during cardiomyocyte isolation was as outlined above with no added Ca^2+^.

The protocol for isolation of cardiomyocytes was as described previously (26). Briefly, the whole heart was rapidly excised and placed into cold, nominally Ca^2+^-free Tyrode's solution (NT). The heart was then cannulated via the aorta on a Langendorff type apparatus and warmed NT buffer at 37 °C was perfused through the heart in a retrograde fashion for 6 min to clear residual blood. The solution was then exchanged for a Ca^2+^-free NT buffer with enzyme mix (containing 15 mg of collagenase type II (Worthington), with 50 mg of bovine serum albumin prepared from Cohn fraction V albumin and 18 mg of protease (type XIV 15% Ca^2+^) in 30 ml) for 8–15 min. Identification of rod-shaped cardiomyocytes in the perfusate was used as an indication of digestion being complete. The solution was then exchanged for a 2 mm Ca^2+^ NT solution, the heart was cut down, and cardiomyocytes were mechanically dispersed from the tissue in a shaking water bath. Typically this method yielded 70–90% rod-shaped cardiomyocytes, which were stored in NT solution at room temperature and used within 18 h of isolating. In intact myocytes, cells were exposed to ZnCl_2_ in the presence of the zinc ionophore, zinc pyrithione (ZnPy) to enable the entry of Zn^2+^ into the cell because ZnCl_2_ is cell-impermeable.

##### Cell Permeabilization

Isolated cardiomyocytes were permeabilized using saponin. Briefly, cardiomyocytes were perfused with 0.5 mm Ca^2+^ Tyrode's solution for 2 min prior to perfusion with a solution containing 100 μm EGTA, 5 mm ATP, 10 mm HEPES, 150 mm potassium gluconate, 25 μm MgCl_2_ at 20 °C. The sarcolemmal membrane was permeabilized with saponin (50 μg/ml) for 30 s. After permeabilization, cardiomyocytes were maintained in recording solution containing 1 mm EGTA, 10 mm HEPES, 120 mm potassium gluconate, 5 mm ATP, 1 mm pyruvate, 1 mm free MgCl_2_, and 100 nm free Ca^2+^ (calculated using the MaxChelator program), and 30 μm Fluo-4, pH 7.2. 1 mm BAPTA was added as indicated in the text, and additional Zn^2+^ was added to maintain as required.

##### Imaging

Images from permeabilized cardiomyoyctes were recorded using an Olympus 1X81 confocal microscope system using Olympus Fluoview software (version 4.0). Line scans were recorded using a 60× oil immersion lens using 2 × 2 pixel binning where each pixel was 0.43 μm in diameter. Fluo-4 indicator was excited using a 488-nm laser, and line scans were acquired at a rate of 333 Hz. The data were analyzed offline using ImageJ software. In our Tyrode's solution alone, in the presence of 100 nm Ca^2+^, there was no significant change in Fluo-4 fluorescence with increasing concentrations of Zn^2+^ (ΔFluo-4 *F*/*F*_0_ 0.026 ± 0.01 units in 10 nm Zn^2+^ compared with control Tyrode's solution with no ZnCl_2_ added). For the cardiomyocyte experiments, ZnCl_2_ stocks were made directly in Tyrode's solution because the maximum concentration of Zn^2+^ used was 100 nm. For measurements of Ca^2+^ or Zn^2+^ in intact cardiomyocytes using Fluo-4-AM or Zinpyr-1, cells were illuminated with 488 nm using a monochromator (Photon Technology International, Birmingham, NJ) with fluorescence emissions captured above 520 nm using a Cascade 512B CCD camera (Photometrics, Tuscan, AZ) and continuously perfused at 32 ± 2 °C. The data were acquired using EasyRatioPro software (Photon Technology International, Birmingham, NJ) and analyzed using Winfluor v3.6.8 software (John Dempster, University of Strathclyde, Glasgow, UK).

##### Contractile Measurements

Spontaneous contractions were measured from cardiomyocytes perfused with Tyrode's solution at 32 ± 1 °C where videos were recorded to DVD. Analysis of contractions was carried out using edge detection measurements in Winfluor version 3.6.8 software.

##### Statistics

The data were expressed as means ± S.E. Where appropriate, a Student's *t* test was used to assess the difference between mean values. A *p* value of 0.05 was taken as significant. Where multiple treatments were compared, ANOVA followed by a Bonferroni post hoc test was used to assess the difference between treatments. A *p* value of 0.05 was taken as significant.

## Results

### 

#### 

##### Effect of Zn^2+^ on RyR2 Function

**FIGURE 1.**
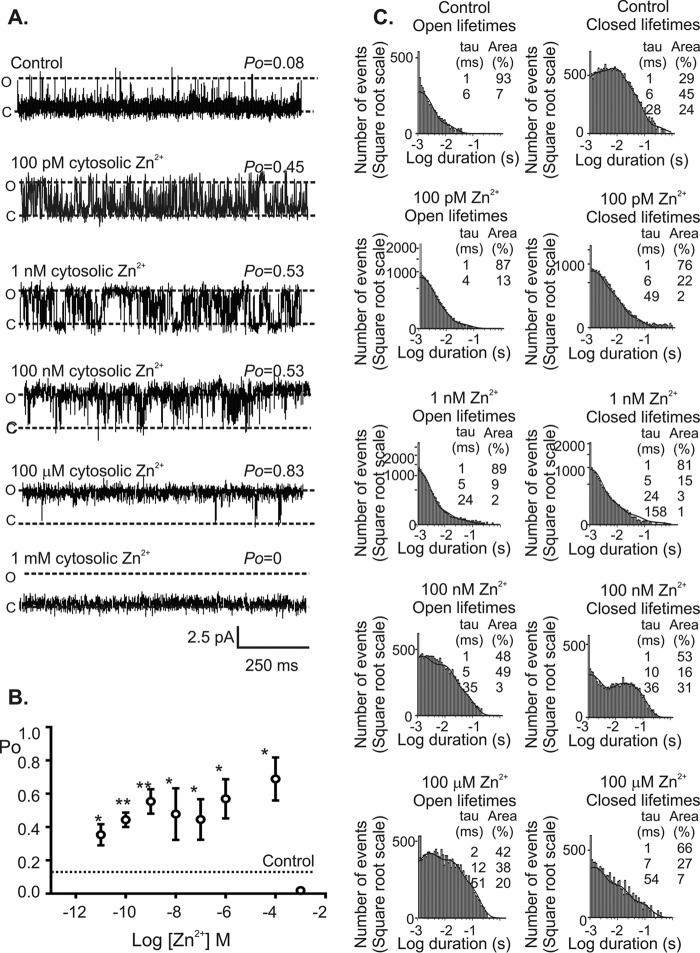
**The effect of Zn^2+^ on RyR2 gating.** *A*, a typical single RyR2 channel showing the effect of sequential additions of Zn^2+^ to the *cis*-chamber (cytosolic face of the channel) in the presence of 10 μm activating Ca^2+^ with Ca^2+^ as permeant ion at a holding potential of 0 mV. Open and closed states are shown by *O* and *C*, respectively. *B*, the relationship between Zn^2+^ concentration and RyR2 *P*_o_. *Error bars* show the mean values ± S.E. (*n* = 5; *, *p* < 0.05). *C*, the effects of Zn^2+^ on open and closed lifetime distributions of RyR2. Open (*left*) and closed (*right*) lifetime distributions and probability density functions of a typical RyR2 channel in the presence of 10 μm cytosolic Ca^2+^ alone (control) and after sequential addition of 100 pm, 1 nm, 100 nm, and 100 μm Zn^2+^ to the cytosolic face of the channel. The best fits to the data were obtained by maximum likelihood fitting. Time constants and percentage areas are shown for each distribution.

**FIGURE 2.**
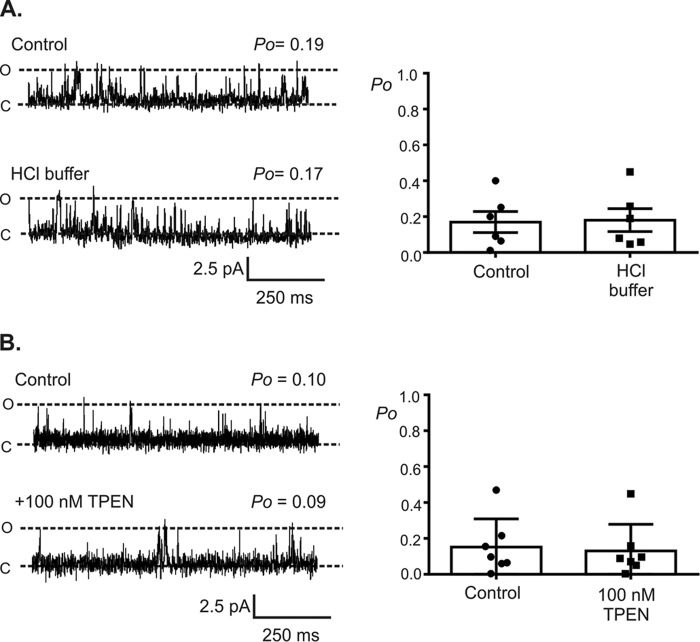
**HCl buffer and TPEN have no effect on RyR2 gating.** The *top traces* in *A* and *B* show representative single RyR2 channel current fluctuations using 10 μm Ca^2+^ as the sole activating ligand. The bilayer was voltage-clamped at 0 mV. The *lower traces* in *A* and *B* show that addition of the HCl buffer (*A*) or the addition of 100 nm TPEN (*B*) to the *cis*-chamber had no significant effect on channel *P*_o_. The open and closed channel levels are denoted by *O* and *C*, respectively. The *bar charts* in *A* and *B* show both the average *P*_o_ and the individual data points following the addition of the HCl buffer (*A*, *n* = 6) or 100 nm TPEN (*B*, *n* = 7). The data are expressed as means ± S.E.

To determine whether Zn^2+^ can alter RyR2 gating, single RyR2 channels were incorporated into phospholipid bilayers enabling the membrane environment to be carefully controlled, and the direct action of Zn^2+^ at the cytosolic face of the channel was studied. Using Ca^2+^ as a permeant ion and holding at a command potential of 0 mV, which is considered to be the resting membrane potential of the sarcoplasmic reticulum (27), the addition of 100 pm Zn^2+^ to the cytosolic face of RyR2 channels significantly increased channel *P*_o_ from 0.10 ± 0.03 to 0.45 ± 0.04 (Fig. 1), indicating that RyR2 has high affinity for Zn^2+^. The potentiation of RyR2 gating by physiological concentrations of free Zn^2+^ suggests that the amount of Ca^2+^ released through a single RyR2 channel following activation by cytosolic Ca^2+^ is significantly greater than previously thought. When Zn^2+^ levels were incremented in a cumulative fashion, channel activation plateaued at 1 nm Zn^2+^, and higher concentrations of Zn^2+^ (>10 nm) had no further significant effect on channel *P*_o_ (Fig. 1*B*). Physiological concentrations of Zn^2+^ will therefore play a key role in regulating graded Ca^2+^ release events via RyR2 channels, enabling flexibility in the force of cardiac contraction, permitting the heart to adjust to altered diastolic filling (28). The addition of 1 mm Zn^2+^ to the cytosolic face of RyR2 completely abolished all channel openings (Fig. 1, *A* and *B*), possibly a consequence of Zn^2+^ binding to the low affinity divalent inhibitory site of RyR2 (29). Control HCl buffer solution containing no Zn^2+^ had no significant effect on RyR2 gating (Fig. 2*A*). To confirm that the level of free Zn^2+^ in our recording solutions, which was measured at ∼9 pm, was insufficient to influence channel gating, we exposed RyR2 channels to the potent Zn^2+^ chelator TPEN. In the absence of exogenously added Zn^2+^, the addition of 100 nm TPEN to the *cis*-chamber caused no significant change in channel *P*_o_ (Fig. 2*B*).

##### Mechanisms by Which Zn^2+^ Regulates RyR2 Gating

To investigate the mechanisms underlying RyR2 activation by Zn^2+^, we performed lifetime analysis on experiments where only a single RyR2 channel was gating in the bilayer. Dwell time histograms of the apparent open and apparent closed states uncovered three modes of Zn^2+^ operation at the cytosolic face of RyR2 (Fig. 1*C*). Control channel-gating was best characterized by short openings and long closing as described previously (30, 31). The addition of Zn^2+^ in the concentration range 100 pm to 10 nm appeared to sensitize RyR2 to cytosolic Ca^2+^, revealed by a reduction in the duration of all the closed states with little effect on the duration of channel openings (32). When the free Zn^2+^ concentration was elevated to >1 nm, RyR2 dwelled for longer sojourns in the open state and displayed briefer sojourns in the closed state (see Table 1 for average lifetime data). This type of gating is characteristic of Ca^2+^-independent channel openings (21).

**TABLE 1:** **Comparison of the changes in RyR2 channel gating in response to Zn^2+^** The table shows the open and closed lifetime parameters in the presence of 10 μm cytosolic Ca^2+^ and following the addition of Zn^2+^ (100 pm to 100 μm) to the cytosolic face of the channel. The values are means ± S.E. (*n* = 3).

[Zn^2+^]	T1		T2		T3		T4	
	Time	Area	Time	Area	Time	Area	Time	Area
	*ms*	%	*ms*	%	*ms*	%	*ms*	%
**Open lifetime parameters**								
Control	1.0 ± 0.05	89.0 ± 3.0	5.0 ± 0.9	11.0 ± 3				
100 pm	1.0 ± 0.1	88.0 ± 1.2	4.6 ± 0.6	12.0 ± 1.0	15.0 ± 8.0	2.0 ± 1.0		
1 nm	1.0 ± 0.1	62.9 ± 9.0	4.5 ± 0.78	32.0 ± 8.0	27.0 ± 4.0	4.6 ± 2.5		
100 nm	1.2 ± 0.3	55.0 ± 14.0	5.2 ± 0.84	34.0 ± 6.0	21.2 ± 4.5	10.6 ± 9.0		
100 μm	2.2 ± 0.4	46.5 ± 0.9	10.2 ± 3.6	43.0 ± 8.0	60.6 ± 20	10.2 ± 6.8		

**Closed lifetime parameters**								
Control	1.1 ± 0.1	85.8 ± 3.9	5.9 ± 0.9	39.3 ± 0.5	25.3 ± 1.8	24.6 ± 6.3	170.0 ± 12.7	6.8 ± 3.1
100 pm	1.0 ± 0.1	72.0 ± 2.3	5.3 ± 0.6	26.0 ± 2.6	46.0 ± 15.0	2.3 ± 0.3		
1 nm	1.0 ± 0.0	75.0 ± 5.3	6.0 ± 1.7	21.6 ± 5.5	42.5 ± 13.4	3.0 ± 1.0	184.0 ± 27.0	1.3 ± 0.6
100 nm	1.1 ± 0.1	48.0 ± 19.8	4.7 ± 1.2	36.5 ± 3.7	31.1 ± 1.3	15.0 ± 18.0		
100 μm	1.0 ± 0.1	62.3 ± 11.1	6.4 ± 2.4	30.3 ± 6.5	41.7 ± 17.2	7.3 ± 5.1		

**FIGURE 3.**
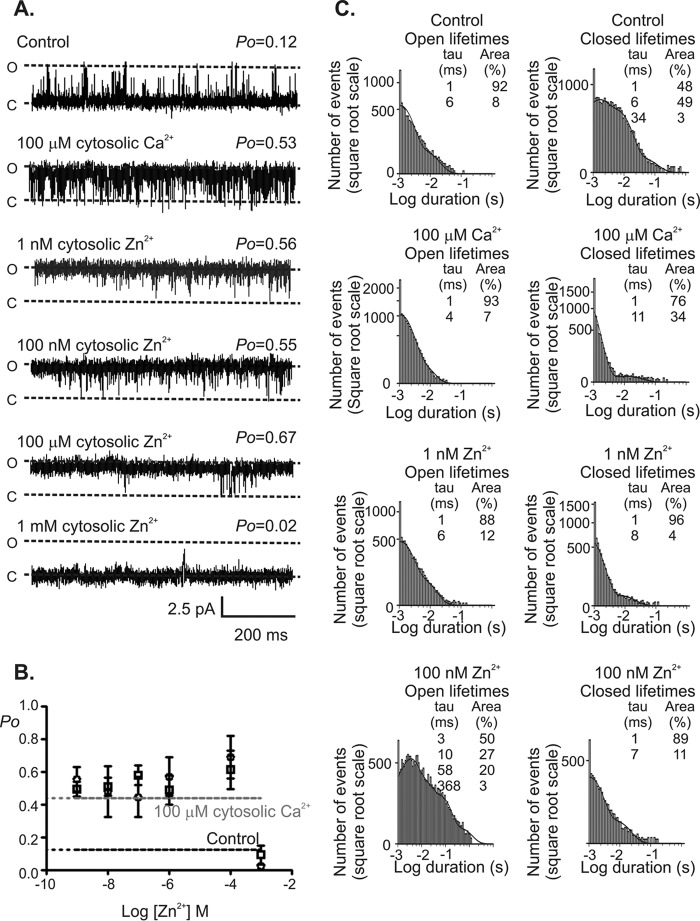
**The effect of Zn^2+^ on RyR2 gating in the presence of a peak levels of Ca^2+^.** *A*, subsequent effect of Zn^2+^ on RyR2 activation following exposure of the channel to 100 μm Ca^2+^ with Ca^2+^ as permeant ion, at a holding potential of 0 mV. *O* is the open state, and *C* is the closed state. *B*, the relationship between RyR2 *P*_o_ and the concentration of Zn^2+^ in the continued presence of 10 μm Ca^2+^ (*open squares*) or 100 μm Ca^2+^ (*open circles*) is shown. The average *P*_o_ using 10 μm (*black dotted line*) or 100 μm Ca^2+^ (*gray dotted line*) as the sole ligand is shown for comparison. *Error bars* show the mean *P*_o_ value ± S.E. (*n* = 5). *C*, open (*left*) and closed (*right*) lifetime distributions for the channel shown in *A* in the presence of 10 μm cytosolic Ca^2+^ alone (control), after raising the cytosolic Ca^2+^ to a peak concentration of 100 μm and following the sequential addition of 1 and 100 nm Zn^2+^ to the cytosolic face of the channel in the continued presence of 100 μm Ca^2+^. The best fits to the data were obtained by maximum likelihood fitting. Time constants and percentage areas are shown for each distribution.

It is clear from the presented data that activating Zn^2+^-binding sites exist on the cytosolic face of RyR2. To assess whether Zn^2+^ binds to the same cytosolic activation sites on RyR2 as Ca^2+^, we first exposed RyR2 to a concentration of cytosolic Ca^2+^ known to elevate *P*_o_ to peak levels (100 μm) (30) and then added subsequent cumulative doses of Zn^2+^ to the cytosolic face of the channel (Fig. 3). In the presence of 100 μm cytosolic Ca^2+^ and using Ca^2+^ as permeant ion, subsequent addition of cytosolic Zn^2+^ in the range 1 nm to 100 μm had no significant effect on RyR2 *P*_o_. Lifetime analysis, however, showed that at concentrations of Zn^2+^ > 1 nm channel gating was altered, and the channel dwelled in longer-lived open states, consistent with Ca^2+^-independent channel openings (21). These data suggest that at least some of the Zn^2+^-binding sites are distinct from the Ca^2+^-binding sites. Irrespective of whether the concentration of cytosolic Ca^2+^ was 10 or 100 μm, the addition of 1 mm cytosolic Zn^2+^ was always found to abolish channel openings. This suggests that in addition to high affinity Zn^2+^ activation sites, RyR2 also has low affinity Zn^2+^ inhibition sites.

**FIGURE 4.**
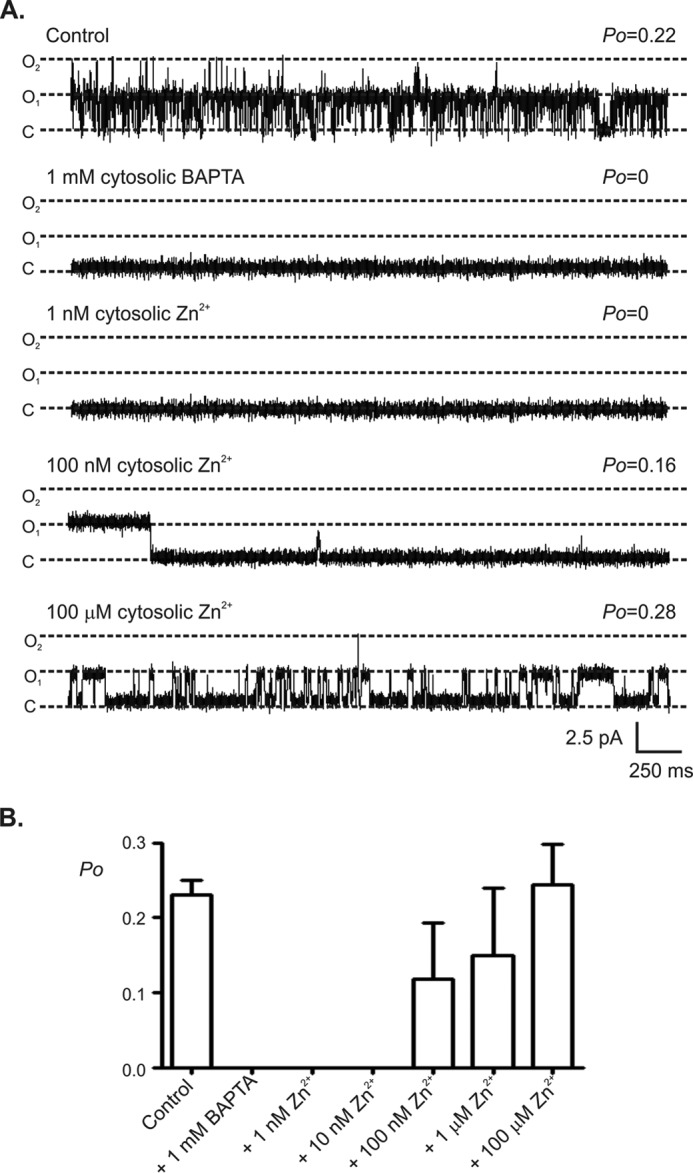
**The effect of Zn^2+^ on RyR2 gating at a subactivating concentration of Ca^2+^.** The *top trace* in *A* shows a typical single RyR2 channel with cytosolic Ca^2+^ as the sole activating ligand and using Ca^2+^ as the permeant ion. The bilayer was voltage-clamped at 0 mV. Subsequent addition of 1 mm BAPTA lowers the Ca^2+^ concentration to <10 nm, which is subactivating, and as expected the channel closes. Subsequent addition of 1 nm Zn^2+^ to the cytosolic face of the channel has no effect, and the channel remains closed. Subsequent addition of 100 nm Zn^2+^ to the cytosolic face of the channel causes the channel to open. These openings are independent of Ca^2+^. Further elevation in the Zn^2+^ concentration causes further channel activation. The open and closed states are denoted by *O* and *C*, respectively. The mean data in *B* show that there is a critical concentration of Zn^2+^ (10 nm), above which RyR2 openings are independent of the cytosolic Ca^2+^ concentration and channel openings are regulated solely by Zn^2+^. The data show the mean values ± S.E. (*n* = 5).

We next examined the interplay between Zn^2+^ and Ca^2+^ at the cytoplasmic face of RyR2 (Fig. 4). Using Ca^2+^ as permeant ion and holding at a command potential of 0 mV, when the free Ca^2+^ was reduced to a subactivating concentration (<10 nm) by the addition of 1 mm BAPTA, as expected, the *P*_o_ value for RyR2 reduced to zero (31). The subsequent addition of 1 nm Zn^2+^ to the cytosolic face of the channel had no effect on *P*_o_, and the channel remained closed because under these conditions, channel gating is dependent on activation by cytosolic Ca^2+^. The subsequent addition of Zn^2+^ in the range 100 nm to 100 μm, however, resulted in channel activations. This is important because RyR2 is now gating in a Ca^2+^-independent manner, whereby Zn^2+^ is the sole activating ligand and the need for Ca^2+^ to activate the channel is removed.

##### Effects of Cytosolic Zn^2+^ on Calcium Waves in Cardiac Cells

**FIGURE 5.**
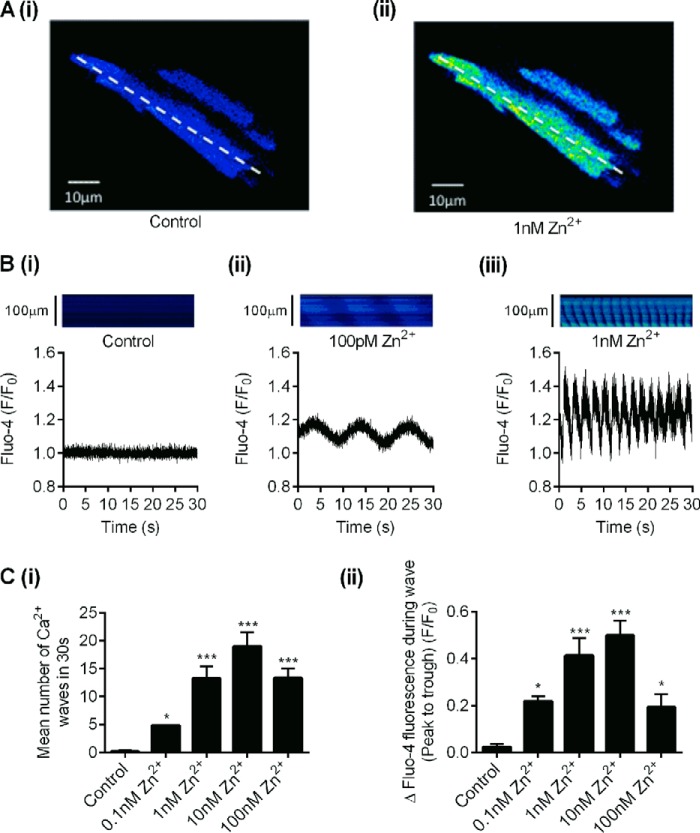
**The effect of intracellular Zn^2+^ on the frequency and amplitude of spontaneous Ca^2+^ waves in isolated cardiomyocytes.** *A*, *panels i* and *ii* show a representative image of Fluo-4 fluorescence in a cardiomyocyte in control and 1 nm Zn^2+^ solution. *B*, a confocal line scan recording taken from a 5-pixel region of interest of Fluo-4 fluorescence from a single cardiomyocyte in control (*panel i*), 0.1 nm Zn^2+^ (*panel ii*), and 1 nm Zn^2+^ (*panel iii*). Confocal line scan recorded from the *dotted line* as indicated in *A. C*, the bar charts show the mean number of Ca^2+^ waves (*panel i*) and the mean amplitude of the waves from cardiomyocytes with increasing concentrations of Zn^2+^ (*panel ii*). (*, *p* < 0.05; ***, *p* < 0.001; one-way ANOVA with Bonferroni's post-hoc test, *n* ≥ 6 cardiomyocytes per data set).

We then measured the effect of Zn^2+^ on Ca^2+^ waves recorded in isolated cardiomyocytes to provide evidence for the modulation of RyR2 function by Zn^2+^ in a cellular environment (Fig. 5). In these experiments, cardiomyocytes were permeabilized to allow the intracellular concentration of Zn^2+^ to be controlled, and Ca^2+^ fluorescence measurements were made using Fluo-4. In control recording conditions, where cardiomyocytes were perfused with an intracellular solution containing 100 nm free Ca^2+^, there were no appreciable change in Ca^2+^ release from the intracellular stores. Perfusion with increasing concentrations of Zn^2+^ (between 100 pm and 10 nm Zn^2+^), however, caused a marked increase in both the incidence of spontaneous Ca^2+^ waves and the amplitude of these events in a Zn^2+^ concentration-dependent manner (Fig. 5*C*). Increasing the Zn^2+^ concentration to >100 nm caused a marked decrease in the amplitude and frequency of waves, and the cardiomyocytes entered an irreversible series of Ca^2+^ waves often culminating in hypercontracture resulting in a loss of membrane integrity. This concentration of Zn^2+^ is close to the pathophysiological concentration of Zn^2+^ previously reported (∼30 nm) (33–36).

**FIGURE 6.**
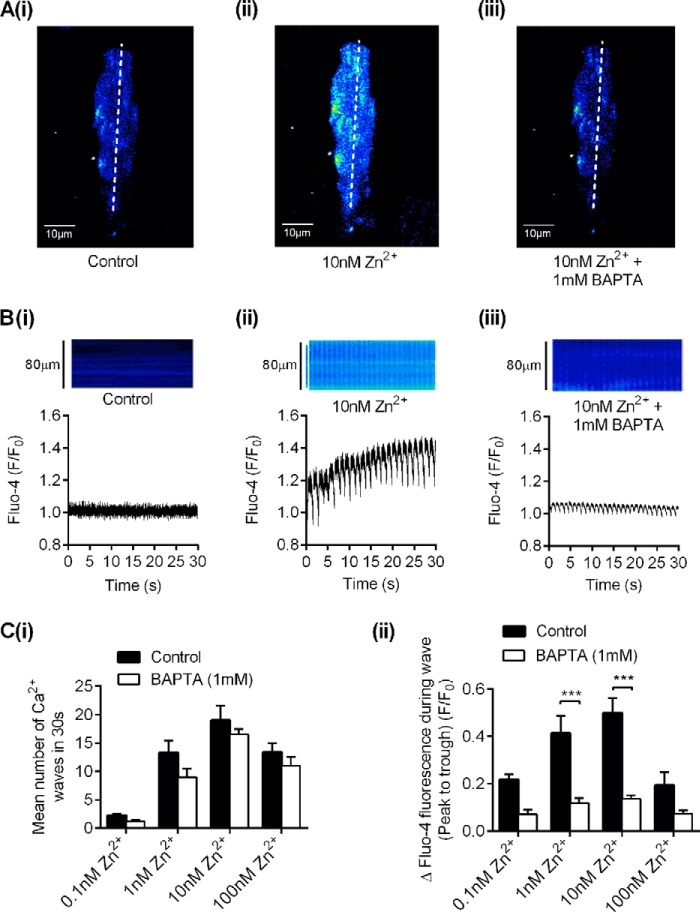
**The effect of buffering intracellular Ca^2+^ with BAPTA on wave amplitude and frequency in isolated cardiomyocytes.** *A*, an example image of an isolated cardiomyocyte in control (*panel i*), in 10 nm Zn^2+^ solution (*panel ii*), and in 10 nm Zn^2+^ solution containing 1 mm BAPTA (*panel iii*). *B*, an example confocal line scan and associated fluorescence changes from a 5-pixel region of interest in control (*panel i*), in 10 nm Zn^2+^ solution (*panel ii*), and in 10 nm Zn^2+^ solution containing 1 mm BAPTA (*panel iii*). *C*, *bar charts* compare the mean number of Ca^2+^ waves (*panel i*) and the mean wave amplitude in control and after treatment with 1 mm BAPTA with increasing concentrations of Zn^2+^ (*panel ii*) (***, *p* < 0.001; two-way ANOVA with Bonferroni's post-hoc test, *n* ≥ 6 cardiomyocytes per data set).

**FIGURE 7.**
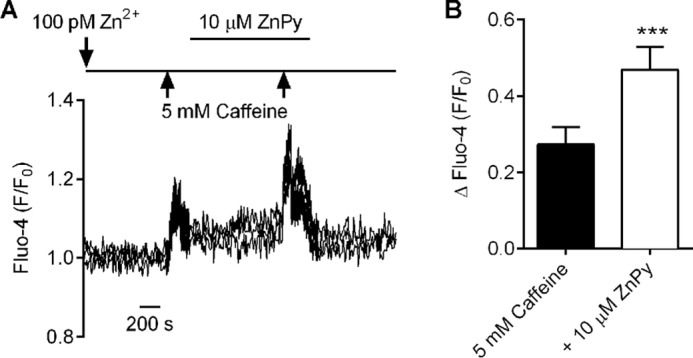
**Elevated intracellular Zn^2+^ levels increase the caffeine-induced Ca^2+^ release.** *A*, example trace showing Fluo-4 fluorescence from three cardiomyocytes perfused with 100 pm Zn^2+^-Tyrode's solution with applications of 5 mm caffeine in the absence and presence of 10 μm ZnPy. *B*, mean change in Fluo-4 fluorescence on application of 5 mm caffeine in 100 pm Zn^2+^-Tyrode's control solution and after application of 10 μm ZnPy (*n* = 4 experiments (66 cardiomyocytes) from 2 animals; ***, *p* < 0.001, paired *t* test).

Interestingly, lowering the concentration of cytosolic Ca^2+^ to subactivating levels while maintaining the concentration of Zn^2+^ did not diminish the Zn^2+^-induced frequency of the Ca^2+^ waves seen in the permeabilized cells. In the presence of 100 nm Zn^2+^, removal of Ca^2+^ by chelation with 1 mm BAPTA markedly reduced the wave amplitude (Fig. 6), presumably as a result of substantial Ca^2+^ buffering. We next examined the caffeine-induced release of SR Ca^2+^ in intact cardiomyocytes in the presence and absence of the ZnPy in Tyrode's solution containing 100 pm Zn^2+^ (Fig. 7). The increase in Fluo-4 fluorescence on application of 5 mm caffeine was markedly potentiated by treatment with 10 μm ZnPy, suggesting that the increase in intracellular Zn^2+^ enhanced the caffeine-induced Ca^2+^ release via RyR2. Consistent with our single channel studies, this shows that Zn^2+^ can directly influence cellular Ca^2+^ dynamics through direct modulation of RyR2. The finding that Zn^2+^ can act as the sole activator of RyR2 highlights a potential pathophysiological consequence of dysregulated Zn^2+^ homeostasis. Such a deleterious cycling of intracellular Ca^2+^ with pathophysiological concentrations of Zn^2+^ could be arrhythmogenic in the intact myocardium.

**FIGURE 8.**
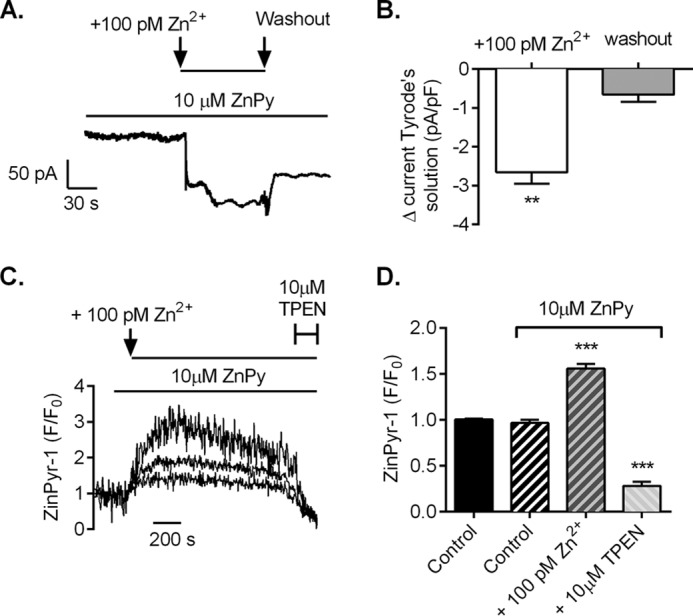
**Elevated extracellular zinc levels result in an increase in the concentration of intracellular zinc.** *A*, example whole cell recording showing the development of an inward current on application of a Tyrode's solution containing an additional 100 pm Zn^2+^ to an isolated cardiomyocyte voltage-clamped at 0 mV. *B*, mean data showing the increase in current from basal in 100 pm Zn^2+^ and washout (*n* = 4 cardiomyocytes from 2 animals; **, *p* < 0.01, repeated-measured ANOVA with Bonferroni's post-test). *C*, example fluorescence recordings from three cardiomyocytes loaded with 10 μm Zinpyr-1 showing that application of 10 μm ZnPy facilitates an increase in intracellular Zn^2+^ when extracellular Zn^2+^ is increased. *D*, mean change in intracellular Zinpyr-1 fluorescence in control, in the presence of ZnPy and after application of TPEN (*n* = 4 experiments (156 cardiomyocytes) from 2 animals; ***, *p* < 0.001; one-way ANOVA with Bonferroni's post-test.).

**FIGURE 9.**
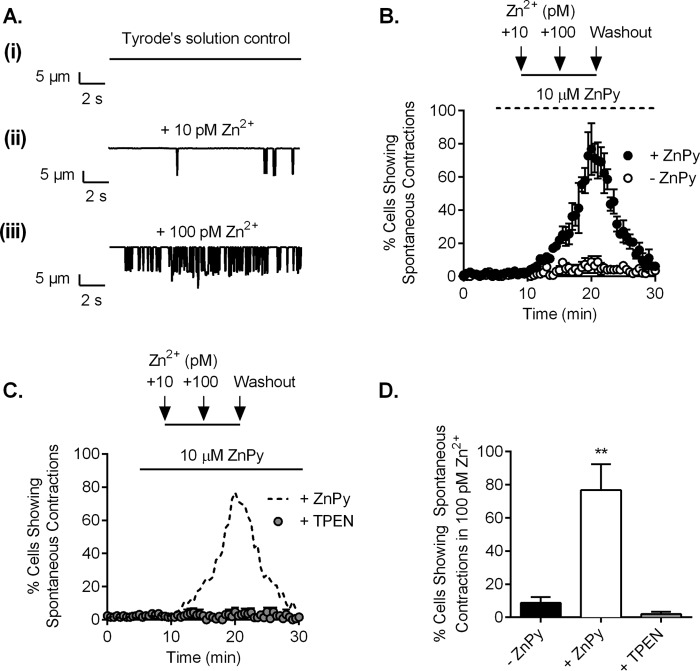
**Elevated intracellular Zn^2+^ can evoke spontaneous contractions in intact cardiomyocytes.** *A*, edge detection measurements from a single cardiomyocyte treated with 10 μm ZnPy in control (*panel i*), with an additional 10 pm ZnCl_2_ (*panel ii*), and with an additional 100 pm ZnCl_2_ showing increasing spontaneous contractions with increasing [Zn^2+^] (*panel iii*). *B*, mean percentage of cardiomyocytes showing spontaneous contractions with increasing [Zn^2+^] in the presence (*black circles*) and absence (*white circles*) of 10 μm ZnPy (*n* = 4 experiments (>100 cardiomyocytes) from 2 animals for each data set). *C*, mean percentage of cardiomyocytes showing spontaneous contractions before (*dashed line*) and after (*gray circles*) pretreatment with 10 μm TPEN. Cardiomyocytes were perfused either with control-Tyrode's solution alone or with an additional 10 or 100 pm Zn^2+^-Tyrode's solution with 10 μm ZnPy. *D*, mean percentage of cells showing spontaneous contractions in 100 pm Zn^2+^ in the absence or presence of 10 μm ZnPy and with 10 μm ZnPy plus 10 μm TPEN (*n* = 4 experiments (>100 cardiomyocytes) from 2 animals for each data set; **, *p* < 0.01; one-way ANOVA with Bonferroni's post-test).

Further evidence to suggest that changes in intracellular Zn^2+^ levels have a marked effect on cellular cardiac contractions comes from raising intracellular Zn^2+^ levels by pretreating intact cardiomyocytes with 10 μm ZnPy and exposing these cells to 100 pm extracellular ZnCl_2_. Under these conditions, there was a significant increase in the inward current carried by Zn^2+^ (Fig. 8, *A* and *B*), and a concomitant increase in intracellular Zn^2+^ measured using the fluorescent indicator Zinpyr-1 (Fig. 8, *C* and *D*). As shown by edge detection measurements, this resulted in spontaneous contractions compared with control cells (Fig. 9, *A* and *B*). The observed spontaneous contractions were reversed by chelating intracellular Zn^2+^ by the addition of 10 μm TPEN (Fig. 9, *C* and *D*), confirming that these cellular effects are attributed to elevated intracellular Zn^2+^ levels.

## Discussion

In this study, we reveal that cytosolic Zn^2+^ is a high affinity activator of RyR2, effective at low picomolar concentrations. Lifetime analysis of RyR2 reveals unique and distinctive gating characteristics dependent on the concentration of cytosolic Zn^2+^. When Zn^2+^ levels are in the range 100 pm to 1 nm, RyR2 activation occurs by sensitization of the channel to cytosolic Ca^2+^. When the concentration of Zn^2+^ is elevated above 1 nm, the effect of Zn^2+^ overrides the influence of cytosolic Ca^2+^, and removal of activating levels of cytosolic Ca^2+^ no longer influences channel *P*_o_. When Zn^2+^ is elevated to toxic levels (in the millimolar range), it inhibits RyR2 channel function. Further evidence for the modulation of RyR2 function by Zn^2+^ in isolated permeabilized cardiomyocytes was acquired through measurement of Ca^2+^ waves. In these recordings, spontaneous Ca^2+^ waves were markedly increased in both frequency and amplitude in a Zn^2+^ concentration-dependent manner (between 100 pm and 10 nm Zn^2+^). Removal of Ca^2+^ by chelation with 1 mm BAPTA did not abolish Ca^2+^ waves in the presence of Zn^2+^, showing that Zn^2+^ can directly influence Ca^2+^ dynamics. These findings suggest an important pathophysiological consequence of dysregulated Zn^2+^ homeostasis, whereby even a small increase in RyR2 open probability during diastole can have major consequences for normal cardiac function and the generation of arrhythmias (37).

It is well established that micromolar concentrations of cytosolic Ca^2+^ are required to activate RyR2 (38, 39). The potentiation of RyR2 activity by physiological concentrations of Zn^2+^ that we now show suggests that Zn^2+^ plays a key role in shaping intracellular Ca^2+^ responses in cardiac muscle. Tight regulation of intracellular Zn^2+^ will therefore be required for the controlled release of Ca^2+^ from SR stores because very small changes in the concentration of free Zn^2+^ will modify the opening of RyR2 channels in response to very small rises in cytosolic Ca^2+^, and this may cause channels to become leaky during diastole. Under pathophysiological conditions, where Zn^2+^ homeostasis is disturbed and levels of Zn^2+^ may reach >1 nm, we propose that Zn^2+^ becomes the main activating ligand of RyR2. Under these conditions, RyR2 starts to gate in exceptionally long open states. The ability of 1 nm Zn^2+^ to activate RyR2 reveals that Zn^2+^ is a high affinity activator of RyR2 displaying approximately 3 orders of magnitude higher affinity for RyR2 than Ca^2+^. This alters the current model of cardiac excitation-contraction coupling where it is accepted that a transient changes in the concentration of intracellular Ca^2+^ is the only trigger for SR Ca^2+^ release and reveals a new role for Zn^2+^ in shaping intracellular calcium responses in cardiac muscle.

Determination of the exact levels of intracellular Zn^2+^ under physiological and pathophysiology conditions is an ongoing area of research, but the development of more sensitive Zn^2+^ probes that are able to measure low concentrations of free Zn^2+^ in single living cells is rapidly advancing (40). At reported pathophysiological levels of cytosolic Zn^2+^ (∼30 nm) (33–36), we show that increases in RyR2 activity are the result of large increases in the lifetime duration of the open channel state rather than an increase in the frequency of openings and this is characteristic of a Ca^2+^-independent component of channel gating. In the presence of ≥10 nm free Zn^2+^, we show that chelation of cytosolic Ca^2+^ is no longer able to close RyR2 channels. This is because there is sufficient Zn^2+^ present to directly open the channel. The balance of intracellular Zn^2+^ is therefore crucial in switching RyR2 channels from a “Ca^2+^-dependent” to a “Ca^2+^-independent” mode of gating.

Our mechanistic approach has for the first time revealed that Zn^2+^ is a potent activator of RyR2. Previous studies in which cardiac muscle [^3^H]ryanodine binding assays were used to assess the effects of Zn^2+^ on RyR2 function failed to see any effect of Zn^2+^ between 10 nm and 1 μm in the presence of 50 μm activating Ca^2+^ (41). This is not surprising because our data now reveal that in the presence of peak levels of Ca^2+^, the effect of Zn^2+^ on channel *P*_o_ is masked. Only by lifetime analysis of our single channel data could we reveal that the mode of channel gating in the presence of Zn^2+^ (>1 nm) was altered and that this type of gating was consistent with Ca^2+^-independent openings. Our single channel data also provide the first evidence that when the concentration of Zn^2+^ is elevated above 1 nm, RyR2 becomes directly activated by Zn^2+^, and the dependence of Ca^2+^ on channel activation is abolished.

It is notable that previous studies have shown a slow accumulation of intracellular Zn^2+^ following prolonged exposure of cardiomyocytes to micromolar levels of extracellular Zn^2+^ and that this results in a reduced SR Ca^2+^ load (42). One plausible explanation for these findings, based on our current data, is that an elevation in intracellular Zn^2+^ removes the Ca^2+^ dependence of RyR2 causing channels to gate in exceptionally long-lived open states. Further evidence for this is demonstrated in intact cardiomyocytes where, in a 100 pm Zn^2+^-Tyrode's solution, the caffeine-induced elevation in intracellular Ca^2+^ was potentiated only after application of 10 μm ZnPy. Potentially this will lead to inappropriate Ca^2+^ release from the SR and likely result in reduced SR Ca^2+^ load. Such a mechanism would be particularly problematic under conditions where levels of intracellular Zn^2+^ are chronically elevated, including diabetes (36) and certain models of dystrophy (43). In line with previous studies (41, 44), we also demonstrated an inhibitory action of Zn^2+^ on RyR2 gating when cytosolic Zn^2+^ is raised to nonphysiological and toxic millimolar concentrations. It is possible that the inhibitory action of Zn^2+^ on RyR2 may be a consequence of Zn^2+^ binding to the low affinity unspecific divalent inhibitory site of RyR2 (29, 45).

Aberrant Zn^2+^ homeostasis has been shown to be associated with cardiomyopathy (46–48), but the underlying mechanism of how Zn^2+^ contributes to cardiac pathology is unknown. Chronic oxidative stress is a major contributor in the pathophysiology of heart failure and under these conditions intracellular levels of Zn^2+^ increase by as much as 30-fold (36). This increase in Zn^2+^ is primarily the result of altered metallothionein function, which perturbs its binding of Zn^2+^, resulting in raised levels (49, 50). Also, recently, a polymorphism in a human gene coding for a member of the metallothionein family has been discovered, and individuals with diabetes who carry this polymorphism have been shown to be more likely to develop cardiovascular complications including chronic heart failure and myocardial damage (51). Diabetic cardiomyopathy is characterized by dysregulation of intracellular Ca^2+^ release, which consequently reduces cardiac contractility and significantly prolongs the rise of systolic Ca^2+^ leading to chronic heart failure (16). The controlled release of Ca^2+^ from the SR during cardiac excitation-contraction coupling is known to govern contractility of the heart. RyR2 plays a fundamental role as the main pathway for the release of Ca^2+^ and drives cellular contraction. Consequently dysfunction in the release of Ca^2+^ and modulatory influences that control RyR2 function are identified as contributory to the pathophysiology of heart failure and fatal cardiac arrhythmias (1). The presented study therefore provides a mechanistic explanation as to how raised levels of intracellular Zn^2+^ may contribute to the progression of contractile dysfunction and heart failure through the alteration of RyR2 gating, which will result in perturbed Ca^2+^ release.

**FIGURE 10.**
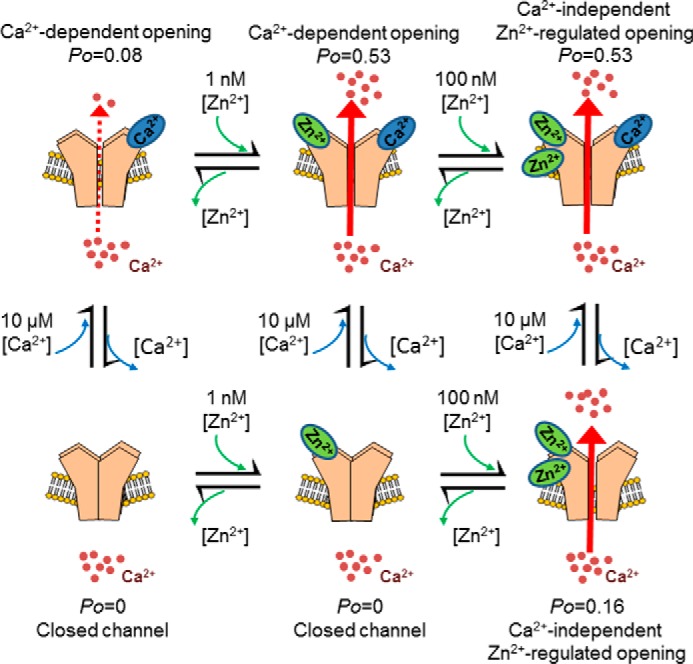
**Proposed model of how Zn^2+^ regulates RyR2-mediated Ca^2+^ release.** When 10 μm cytosolic Ca^2+^ is the sole ligand, channel openings are brief, and the channel gates with a low *P*_o_. Addition of 1 nm Zn^2+^ to the cytosolic face of the channel causes an increase in the channel *P*_o_, and the channel gates with high frequency openings. In both of these conditions, RyR2 gating is regulated by cytosolic Ca^2+^, and lowering the concentration of free Ca^2+^ to subactivating levels (<10 nm) reduces RyR2 *P*_o_ to 0. If the Zn^2+^ concentration is elevated above 1 nm, RyR2 becomes uncoupled from the usual regulatory effects of cytosolic Ca^2+^, and Zn^2+^ becomes the sole activating ligand.

A simple model to explain how cytosolic Zn^2+^ modulates RyR2 gating is shown in Fig. 10. We suggest that under physiological conditions, cytosolic Zn^2+^ is able to fine-tune the release of Ca^2+^ from the SR while in parallel enabling flexibility and control of SR Ca^2+^ dynamics mediated through RyR2. Under pathophysiological conditions, where Zn^2+^ homeostasis is disturbed and levels of Zn^2+^ may reach >1 nm, RyR2 starts to gate in exceptionally long open states, which leads to very high *P*_o_ potentially leading to arrhythmia. In this conformation, RyR2 is uncoupled from the usual regulatory effects of cytosolic Ca^2+^ and RyR2 channels are now activated by solely by Zn^2+^.

Our study reveals that RyR2-mediated Ca^2+^ homeostasis is intimately related to intracellular Zn^2+^ levels and provides a mechanistic explanation as to how altered Zn^2+^ homeostasis can modulate cardiac RyR2 function. We reveal that Zn^2+^ is a high affinity activator of RyR2 which challenges our understanding of cardiac excitation-contraction coupling. We suggest that under normal physiological conditions, intracellular Zn^2+^ is essential in fine-tuning the release of Ca^2+^ from the SR. Pathological perturbations in Zn^2+^ homoeostasis will lead to inappropriate release of Ca^2+^ as observed in certain cardiac abnormalities including heart failure and fatal arrhythmias.

## Author Contributions

J. W. and R. D. R. performed experiments and analyzed data; A. J. S. designed the experiments and contributed toward writing the manuscript; and S. J. P. designed the experiments, performed experiments, analyzed data, wrote the manuscript, and supervised the project. All authors discussed the results and implications and commented on the manuscript at all stages.

## Footnotes

2 The abbreviations used are:

RyR2: type 2 ryanodine receptor
SR: sarcoplasmic reticulum
BAPTA: 2,2′-(ethylenedioxy)dianiline-*N*,*N*,*N′*,*N′*-tetraacetic acid
TPEN: *N*,*N*,*N′*,*N′*-tetrakis(2-pyridylmethyl)ethylenediamine
ZnPy: zinc pyrithione
ANOVA: analysis of variance.
